# Automated Determination of Bone Age in a Modern Chinese Population

**DOI:** 10.5402/2013/874570

**Published:** 2013-02-25

**Authors:** Shao-Yan Zhang, Gang Liu, Chen-Guo Ma, Yi-San Han, Xun-Zhang Shen, Rui-Long Xu, Hans Henrik Thodberg

**Affiliations:** ^1^Hebei Research Institute of Sports Sciences, 372 Zhongshan East Road, Shijiazhuang, Hebei 050011, China; ^2^Dalian Sports Science Research Institute, 31 Shikui Road, Dalian, Liaoning 116011, China; ^3^Wenzhou Sports Science Research Institute, 22 Youyongqiao Road, Wenzhou, Zhejiang 325000, China; ^4^Shanghai Sports Sciences Research Institute, 87 Wuxing Road, Shanghai 200030, China; ^5^Guangdong Sports Science Research Institute, 818 Aoti Road, Guangzhou, Guangdong 510663, China; ^6^Visiana, Søllerødvej 57 C, 2840 Holte, Denmark

## Abstract

*Rationale and Objective*. Large studies have previously been performed to set up a Chinese bone age reference, but it has been difficult to compare the maturation of Chinese children with populations elsewhere due to the potential variability between raters in different parts of the world. We re-analysed the radiographs from a large study of normal Chinese children using an automated bone age rating method to establish a Chinese bone age reference, and to compare the tempo of maturation in the Chinese with other populations. *Materials and Methods*. X-rays from 2883 boys and 3143 girls aged 2–20 years from five Chinese cities, taken in 2005, were evaluated using the BoneXpert automated method. *Results*. Chinese children reached full maturity at the same age as previously studied Asian children from Los Angeles, but 0.6 years earlier than Caucasian children in Los Angeles. The Greulich-Pyle bone age method was adapted to the Chinese population creating a new bone age scale BX-China05. The standard deviation between BX-China05 and chronologic age was 1.01 years in boys aged 8–14, and 1.08 years in girls aged 7–12. *Conclusion*. By eliminating rater variability, the automated method provides a reliable and efficient standard for bone age determination in China.

## 1. Introduction 

Globalization is not a new trend in auxology. For decades, researchers have compared growth and maturation in populations across the world, exploring racial and regional differences and studying the effects of changes in lifestyle.

In particular, there has been a longstanding interest in the differences in tempo of maturation among populations and their secular trend. Such studies have preferentially been performed using bone age (BA) determinations. Unlike secondary sexual characteristics, BA can reveal the entire course of maturation from birth to adulthood. However, bone age rating is associated with considerable operator variability, and it is particularly difficult to ensure consistency between raters from widely separated parts of the world.

The emergence of a fully automated and therefore rater-independent BA rating method [1] has the potential to overcome this problem and, thereby, revitalize comparative studies of maturation across the world. 

We present a reanalysis of a large study of hand radiographs involving modern, normal Chinese children using this automated bone age system. 

The study could also have considerable impact on clinical practice in China. Due to the one-child policy, parents have a strong focus on growth and development of their child, and currently, there are more than 500.000 BA determinations in China per year. This need is growing, but there is a shortage of persons qualified to do the rating. Adult height prediction in Asian children based on automated BA will be reported in a separate paper. 

Our study had the following objectives:

1. to validate the automated method by assessing its ability to analyse these images and its agreement with manual Tanner-Whitehouse (TW) ratings [2];
2. to derive reference curves for Greulich-Pyle (GP) BA [3] of Chinese children, to study their regional variation in China, and to compare the curves to American children studied with the same bone age method;
3. to construct a Chinese BA scale (called BX-China05) as an adaptation of the GP BA for the Chinese population in 2005;
4. to construct a Chinese TW BA scale called TW-China05.

## 2. Subjects and Methods

### 2.1. Study Data

Left hand radiographs of 2883 males aged 2–20 years and 3143 females aged 2–19 years were extracted from a previous study of children of the *Han* ethnicity from five different cities in China [4]. The full age range was covered in the children from *Dalian*, *Wenzhou*, and *Shijiazhuang*, while the age range started at 5 years in *Guangzhou* and at 7 years in *Shanghai*. The images were taken close to the children's anniversaries; in addition, images were taken at 2.5 and 3.5 years of age (the age distribution can be appreciated from Figure 2). The original study was approved by the Ethical Committee of the Medical Department, Hebei Sports Science Institute, in 2002. Before the study started, a questionnaire was given to children's parents for surveying background such as parent's occupation, education level, income, and the medical history of the children and their family, and a written informed consent was obtained from the parents and/or the children. The children were healthy and representative of the general population in these urban regions.

The films were scanned using a Vidar Diagnostic Pro Advantage scanner (Hemdon, USA) in 150 dpi and 12 bits per pixel. The radiographs formed a representative sample of the previous study which comprised 17,000 radiographs recorded on films. These radiographs had previously been BA rated by the first author using the TW method.

### 2.2. Analysis Method

The images were analysed using the BoneXpert version 2.1 automated method for BA determination (Visiana, Denmark, http://www.BoneXpert.com), which determines GP and TW3 BA [5–7]. The method was developed on Caucasian children and has been validated on Asian children from Japan and California [8, 9]. The TW BA rating, more specifically, the assignment of stages to each bone, was recently recalibrated in order to coincide with ratings from the First Zurich Longitudinal Study [10]. This rating, called standard TW rating, has been shown to be consistent with Tanner's ratings of the so-called Gold Series of reference images. 

In order to compare bone maturation in different regions, reference curves of BA − CA versus chronological age (CA) were derived for each city separately and for all cites combined, using GP BA.

The GP method is the most widely known and used BA rating method. It is fast and reliable, because the image is rated by direct comparison to reference images. However, the GP BA scale refers to mid- to upper-class American Caucasian children from the 1930s, a standard group remote from present-day Chinese children. We therefore developed a variant of GP BA called *BX-China05* as follows. 

The GP BA is considered to be not an estimate of CA, but rather an abstract maturity measure, and for each age, we determined the median GP BA of Chinese children. The relationship between CA and median GP BA was tabulated and used to transform an observed GP BA value into an age value, defined as BX-China05. For example, if the median GP BA is 13.5 at a CA of 13 years, a child with a GP BA of 13.5 has a BX-China05 of 13 years.

This method of mapping a maturity measure to a population-specific bone age is not new; it was originally conceived in the context of the TW method, and we also carried it through for the TW method in this study. Therefore, for each age, we derived the median Sum Maturity Score (SMS) of the automated TW rating in order to derive a table for transforming an observed SMS value into a bone age, which we call *TW-China05 *(such a table had been estimated before using the *manual* TW ratings of these image data [4]. By reestimating the table using *automated* TW ratings, we ensured that the table was consistent with BoneXpert's standard TW rating of bone stages).

## 3. Results

### 3.1. Efficiency

Thirty images were rejected by BoneXpert before arriving at the 6026 images of this study. Most of these rejections were due to poor image quality or a bone age below the lower limit of the intended range of BoneXpert (2.5 years for boys and 2.0 years for girls), but 12 of the rejected images were of good quality, corresponding to 0.2%, so the observed efficiency of the method was 99.8%. 

### 3.2. Agreement of BoneXpert with Human Raters

**Figure 1:**
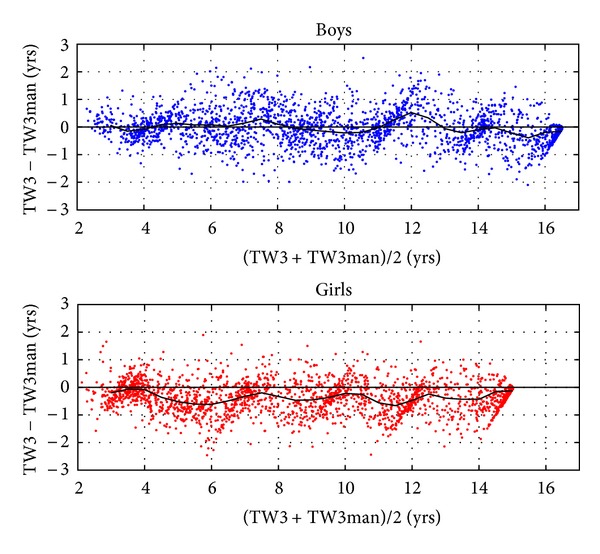
Bland-Altman plot showing the difference between the automated and the manual TW3 bone age ratings of 6062 Chinese children and adolescents. The black curve is a running average.

Figure 1 shows the agreement between the manual TW3 rating and BoneXpert's TW3 rating, expressed as a Bland-Altman plot, that is, the difference is plotted versus TW3mid, the average of the two TW3 bone ages.

Focusing on the TW3mid range of 2.5–16 years for boys and 2.5–14.5 years for girls, the average difference between automated and manual TW3 ratings was 0.00 years for boys and −0.37 years for girls. The root mean square (rms) deviations were 0.64 for boys and 0.68 years for girls in the same TW3mid range.

### 3.3. Reference Curves for Maturation

**Figure 2:**
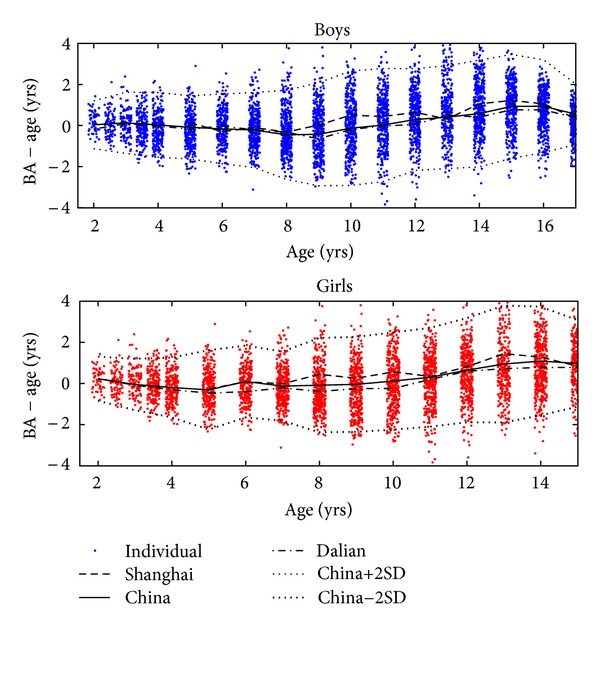
The difference between Greulich-Pyle bone age and chronological age, shown versus age. The data have been spread a bit around the true age for better display. The solid line indicates the average at each age, while the short dashed lines are drawn at the mean value plus or minus two SDs at each age. The Shanghai and Dalian curves are the mean values for these two cities.

To study the difference in tempo of maturation across various populations and ethnicities, we used the GP BA minus the age (CA). Figure 2 displays the observed BA − CA data, and the mean and ±2 SD curves are indicated. The SD for boys within the age range of 8–17 years was 1.26 years, and the SD for girls aged 7 to 15 years was 1.12 years (the SD curve was computed in 1-year intervals, and the quoted SD numbers are the averages over the entire age interval). 

**Figure 3:**
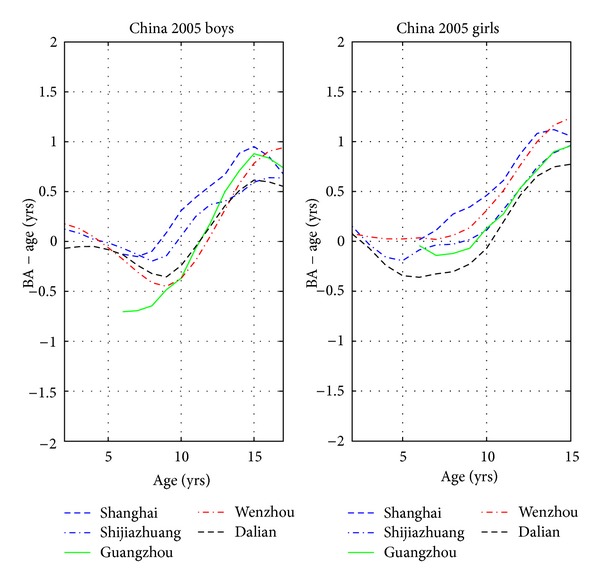
The average Greulich-Pyle bone age minus age for children from five different cities in China.

**Figure 4:**
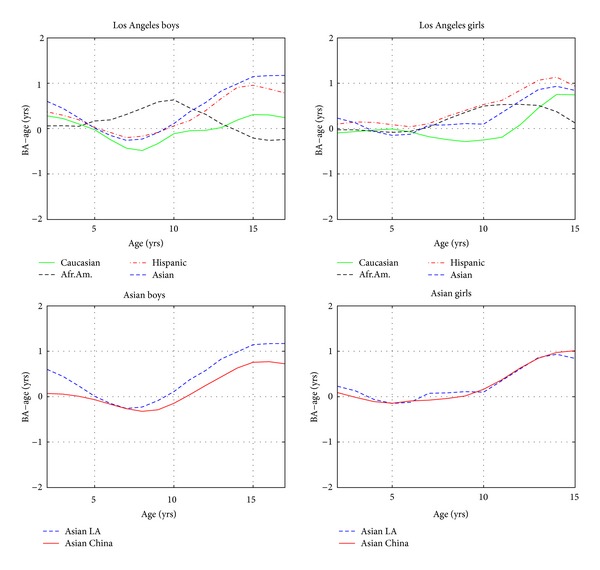
Comparison of Greulich-Pyle bone age minus age for children of various ethnicities. The upper two plots compare four ethnicities in Los Angeles while the lower plots compare Asians in Los Angeles and China.

Figure 3 shows the average curves for each of the five cities, and Figure 4 compares the BA − CA curves with data on four ethnicities from Los Angeles, USA.

### 3.4. The New BX-China05

**Figure 5:**
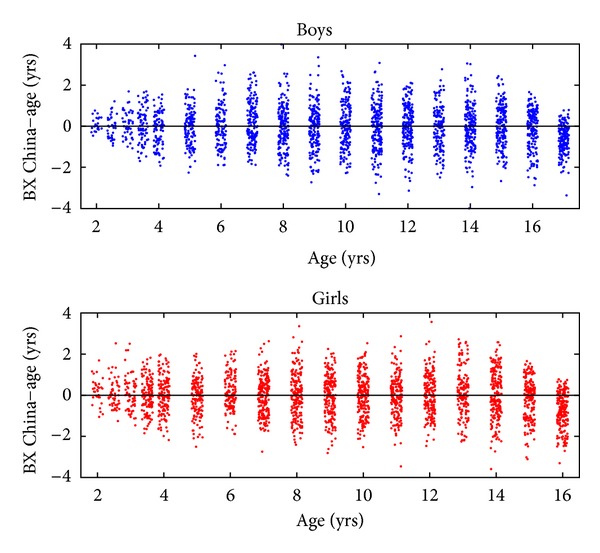
Difference between the new bone age scale BX-China05 and age. BX-China05 is an adaptation of Greulich-Pyle bone age method to Chinese children in 2005.

The new bone age, BX-China05, is reported in Table 1. To calculate BX-China05 for a new image, one should first determine the BoneXpert GP BA and then apply the correction from Table 1. The new BX-China05 was compared with the ages in Figure 5. It is seen that the average difference was close to zero for all ages as expected. The SD for boys over the age range of 8–14 years was 1.08 years, and for girls aged 7–12 years, the SD was 1.01 years.

### 3.5. The New TW-China05

**Figure 6:**
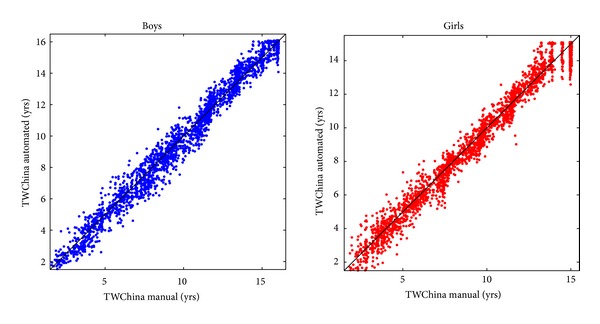
Comparison of manual and automated TW-China05 bone age ratings.

Table 2 shows the relationship between BoneXpert's SMS and TW-China05, the new TW BA adapted to this population, and Figure 6 compares BoneXpert's TW-China05 BA and the manual TW-China05 BA.

The SD of TW-China05 − CA for boys aged 8–14 years was 1.09, and for girls aged 7–12 years, it was 1.01 years. 

## 4. Discussion 

### 4.1. Efficiency

We found that BoneXpert was able to determine bone age for 99.8% of the good-quality images of Chinese children. This result compares well with previous results in Caucasian children, despite that this is a new ethnic group, and the similar performance is probably due to fact that the variation between the average Caucasian and Asian child is much smaller than the variation within the Caucasian population itself.

### 4.2. Agreement with Human Raters

There was good agreement between automated standard TW3 rating and manual TW3 as shown in Figure 1. Firstly, there was only a small average difference (bias) of 0.00 years in the boys and 0.37 years in the girls, which indicates that the Chinese TW rater was largely consistent with the European raters underlying the calibration of BoneXpert's standard TW rating. This agreement was in contrast to that reported in [10] where a Japanese rater had a bias of up to 1.19 years. 

Secondly, the rms deviation (0.64 years for boys and 0.68 years for girls) was smaller than that observed for the Zurich raters [10]. One reason for this slightly better result may lie in the fact that the Chinese children were rated by a single rater while the Zurich images were rated by one of two raters. 

The Chinese rater was never trained by authoritative raters on the TW Gold Series radiographs in London or on other series. He learned the rating from the book [2] but still achieved good agreement with the automated rating. Such an achievement has been reported once before for a Belgian reader [11]. 

### 4.3. Reference Curves for Maturation

Figure 2 shows BA − CA versus age. The most striking aspect is that, at the end of puberty, the average BA − CA has reached approximately 1 year. In other words, Chinese children reach maturity around one year earlier than the children that made up the GP BA reference, in agreement with previous studies [12]. This dramatic deviation was the main motivation for setting up the BX-China05 BA scale.

Figure 2 also shows that the spread in maturity is rather large in Chinese children. This is partly due to the fact that the children were from several cities, separated by large distances. To illustrate this effect, Figure 2 shows the average BA − CA for the two cities at either end of the BA − CA spectrum, Dalian, a middle-size city in the North, and Shanghai, a large city in the south. Our pooling of all five cities contributed to a larger SD of BA − CA. Even within each city, there was a relatively large spread, presumably due to differences between social classes. 

Figure 3 reveals the significant differences between the five cities, with Shanghai being the most advanced in BA and Dalian the most delayed, and the other cities lie in between these two extremes. In principle, these data could be used to set up city-specific reference curves for BA − CA. However, we believe that this would be impractical, and one would be faced with ambiguities when a child moves from one city to another, or if the child is from a city not among the five investigated here. We prefer to pool all children and treat city differences as a statistical uncertainty. A limitation of this sample is that all data come from urban regions and middle and upper social classes, so the sample might not be representative for children from a rural community or children living under poor social conditions.

Figure 4 compares the Chinese BA − CA curves with data on four ethnicities from Los Angeles, USA [9]. This comparison exploits the strength of the automated method. For the first time, one is able to compare maturity of Chinese children with children in the West using a method guaranteed to have no rater bias. The upper part of the figure compares Los Angeles children of different ethnicities, showing that Asians and Hispanics in Los Angeles have similar maturation tempo, while the lower plot compares Asian children from Los Angeles and China. It is interesting that they follow almost the same pattern. Chinese boys lag slightly behind American Asian boys. We see that Asians, averaged over the two sexes, reach maturity 0.6 years before American Caucasians. This comparison of the populations with each other is more interesting than a comparison to the outdated GP standard. In [9], it was reported that American Caucasians reach maturity 0.4 years before Europeans, so we infer that Asians reach maturity a full year ahead of European Caucasians.

### 4.4. The New BX-China05

The new bone age BX-China05 is defined by Table 1, which can conveniently be built into the automated method. The table can also be used to transform a manually determined GP BA to BX-China05. The table can even be used to adjust the ages of each plate of the GP atlas, thereby “translating” it to Chinese. For instance, the female plate with GP BA 12.0 years can be labelled 11.5 years for Chinese females.

The new BX-China scales end 1.2 years earlier than the original GP BA scale.

### 4.5. The New TW-China05

The new TW-China05 scale ends at ages 16.1 years for boys and 15.1 years for girls, similar to the endings of the corresponding manual TW-China05 scale [4] of 16 and 15 years, respectively, as also illustrated in Figure 6.

### 4.6. Discussion of BX-China05 and TW-China05

The BX-China05 and TW-China05 BA scales appear similar. Both are adapted to agree, on average, with the age in the Chinese population. However, there are important differences, because BX-China05 is based on the GP method whereas TW-China05 is based on the TW method. 

GP and TW BA rating represent two rather different methods. When used for manual rating, the TW method has the advantage that it employs an explicit procedure that ensures that the same bones are used, with the same weights, by all raters. The GP method, on the other hand, has the advantage that it easier to learn and can be performed in a fast and intuitive manner. Furthermore, its reliance on a direct comparison of the image with the plates in the atlas ensures that raters are constantly “calibrated” to the correct level. 

The TW method based on manual rating has been used extensively in China [4], rather than the GP method, because the latter refers to Pre-WW II Ohio children which are far removed from modern Chinese children.

This paper has introduced BX-China05 for Chinese children, based on the GP method. The GP atlas is both a *method* and a BA *standard. *With BX-China05, one uses the GP *method* but escapes its BA *standard* by employing the corrections in Table 1. 

With the introduction of automated methods, and the “recalibration” of GP BA in the shape of BX-China05, the balance between the TW and the GP methods changes in favour of the GP method, which has several advantages relative to the TW method.

1. In the automated method used in this paper, the TW and GP methods are both based on the 13 RUS bones (Radius, Ulna, and 11 Short bones) chosen by Tanner for the TW method. The GP method assigns a uniform weight of 7.7% to all 13 bones, while the TW method places a larger weight of 20% on both radius and ulna. Any uncertainty in the interpretation of radius and ulna will, therefore, have a disproportionately large effect on the TW BA, and, indeed, the precision of the automated GP BA is considerably better (0.18 years SD) than that of the automated TW BA (0.30 years) [8].
2. Adult height prediction has been found to be more accurate using GP compared to TW BA, primarily due to the large weight of the radius and ulna in the TW method. The BoneXpert method for adult height prediction is, therefore, based on GP BA (this is also true for the method for Chinese children, presented in a separate paper).
3. The TW3 BA terminates at 16.5 and 15 years for boys and girls, respectively, while the GP BA terminates at 19 and 18 years. In other words, the TW3 method stops 2.5–3 years earlier. This also holds for the automated versions where TW3 and GP agree well up to the BA where TW3 saturates [10]. The same findings are noted in the Chinese version where TW-China05 stops 1.7 years before BX-China05. Therefore, when rating the maturity of adolescents, the GP method is the more powerful method. Notice, however, that the automated method has the general limitation that it becomes more uncertain above a certain maturity level, which is 17 and 15 years of GP BA for boys and girls, respectively. This translates to the limits of 15.8 and 13.8 years, respectively, on the BX-China05 scale. Above these limits, there are only small maturity-related changes in the appearance of the short bones.

In rare cases where the automated method fails, it is recommended to record a new X-ray, unless the child has abnormal bone structure. One can also perform a manual rating using the GP atlas and then use Table 1 to correct the GP BA to the BX-China05. It would even be acceptable to apply the manual TW-China05 method, in case the rater is more familiar with this method.

## 5. Conclusion 

We have presented an important step toward the application of automated BA assessment to Asian populations around the world. We have found that Asian children in Los Angeles, and in mid- to large-sized cities in China, have approximately the same maturation rates.

Asians reach full maturity 1.2 years before the age indicated by the GP BA reference, and, thus, the use of this standard in China is considered misleading by some. On the other hand, as we have pointed out, there are many advantages of the GP atlas methodology, and we have presented an adaptation of the GP BA to Chinese children, called BX-China05. This is recommended for routine clinical use as well as in sports medicine.

## Figures and Tables

**Table 1:** This table is used to transform an observed Greulich-Pyle bone age (GP BA) into the bone age scale BX-China05, adapted to Chinese children in 2005. The Boys column indicates the correction for boys, and similarly for the Girls column. Example: A girl with GP BA = 10 y has BX-China05 = 10 − 0.1 = 9.9 years.

GP BA (years)	BX-China05 Correction (years)	
	Boys	Girls
2.0	0.0	−0.2
2.5	−0.1	−0.2
3.0	−0.2	0.1
3.5	−0.2	0.3
4.0	−0.1	0.4
4.5	−0.0	0.4
5.0	0.0	0.3
5.5	0.1	0.1
6.0	0.2	−0.1
6.5	0.3	−0.1
7.0	0.5	0.2
7.5	0.6	0.3
8.0	0.5	0.2
8.5	0.4	0.1
9.0	0.3	−0.0
9.5	0.2	−0.1
10.0	0.1	−0.1
10.5	0.0	−0.2
11.0	−0.1	−0.2
11.5	−0.2	−0.3
12.0	−0.4	−0.5
12.5	−0.5	−0.6
13.0	−0.4	−0.7
13.5	−0.4	−0.8
14.0	−0.4	−0.9
14.5	−0.5	−1.0
15.0	−0.7	−1.2
15.5	−0.9	−1.2
16.0	−1.0	−1.1
16.5	−1.1	−1.1
17.0 and above	−1.2	−1.2

**Table 2:** The correspondence between TW-China05 bone age and SMS. Example: A boy with SMS = 296 has TW-China05 = 10 years.

TW-China05 Bone age	SMS
Boys	

2.0	79
2.5	98
3.0	117
3.5	123
4.0	134
5.0	156
6.0	180
7.0	209
8.0	239
9.0	262
10.0	296
11.0	345
12.0	426
13.0	517
14.0	642
15.0	835
16.0	959
16.1	1000

Girls	

2.0	162
2.5	174
3.0	188
3.5	201
4.0	210
5.0	231
6.0	264
7.0	306
8.0	347
9.0	420
10.0	496
11.0	593
12.0	700
13.0	861
14.0	956
15.0	986
15.1	1000
